# The 10 Commandments of Open Aortic Arch Repair

**DOI:** 10.1177/15569845221112636

**Published:** 2022-08-02

**Authors:** Paul Werner, Marie-Elisabeth Stelzmüller, Stephane Mahr, Marek Ehrlich

**Affiliations:** 1 Department of Cardiac Surgery, 27271 Medical University of Vienna, Austria

## Introduction

The aortic arch remained the last frontier of aortic surgery until the first successful replacement by DeBakey and Cooley more than 60 years ago.^1^ In the initial years, when arch surgery was in its infancy, it was associated with serious morbidity and mortality. Nowadays, surgical procedures of the arch have become standardized, and satisfying outcomes can be achieved when performed by experts in high-volume centers.^2–4^ This is attributable to improved cardiopulmonary bypass and perfusion strategies, the introduction of the hypothermic circulatory arrest,^5,6^ advancements in neuroprotective strategies,^7–9^ and the implementation of hybrid repair concepts. Nevertheless, arch surgery remains invasive; its goal should be to provide the patient with a long-term solution for the underlying aortic pathology while preventing devastating complications. Several considerations and requirements for successful arch procedures do exist, which are summarized herein as the 10 commandments of open aortic arch repair.

## 1. Have a Dedicated Aortic Team

Aortic arch repair is a surgically demanding procedure; individual perfusion strategies are required, and challenging anastomoses at difficult angles with low-quality aortic tissue are frequently encountered. Analyses from larger patient sets have shown a clear relationship between center volume, surgeon volume, and patient outcomes for thoracic aortic procedures.^10,11^ These data underline the need for a dedicated aortic team, in which each surgeon should have a minimum caseload per year, which can be only ensured in high-volume centers. Furthermore, members of the aortic team should be associated with their patients outside the surgical theater. Patients requiring arch repair often undergo staged procedures. Therefore, a continuity of the treating physician is recommended to provide high-quality, patient-centered care.^12^

The aortic team ideally consists of several specialties, combining surgery with anesthesiology and interventional radiology. Regular meetings to discuss complex procedures (aortic board, similar to heart team meetings) should be established, to offer comprehensive solutions to complex patients. Aortic boards present a unique possibility to acquire expertise in patients with rare aortic and arch pathologies.

## 2. Rely Only on High-Quality Aortic Imaging

Preoperative imaging in open arch repair is mandatory to facilitate optimal procedural planning. Computed tomography angiography (CTA) remains the gold standard for aortic imaging because of its high special resolution and fast image acquisition. We generally recommend, that every patient requiring open aortic arch repair undergoes preoperative CTA (except in salvage situations with acute aortic syndromes). In case of known allergic reactions to contrast agents, we adhere to a preparatory medication regimen including methylprednisolone (by mouth 12 and 2 h pre-scan) and diphenhydramine (intravenously immediately before scan).^13^ CTA for planning of aortic arch repair should adhere to specific standards, which are explained in the following section.

All supra-aortic vessels need to be visible, and ideally, imaging of the cerebral vessels should be performed up to the level of the circle of Willis to allow assessment of the intracranial arterial supply. Clear visualization of the subclavian arteries is mandatory to prevent embolic complications after cannulation in case of severe calcifications or false lumen perfusion in a dissection setting.

The aortic arch and the ascending and descending aorta need to be imaged entirely to correctly prepare for adequate repair. Surgeon experience often allows the correlation of imaging data to expected tissue quality, which can influence the procedural planning (e.g., the location [zone] of the distal anastomosis). Only contrast agent–based CT provides information on the existence and extent of soft plaques in the aortic wall, which is imperative information. One should avoid loss of imaging quality due to movement artifacts; therefore, electrocardiogram gating or fast acquisition protocols with modern scanners are highly recommended. CTA should include the abdominal aorta, the iliac vessels, and the femoral vessels up to the femoral bifurcation to allow for complete assessment of possible cannulation sites.

## 3. Know Which Type of Open Arch Repair is Required (and Feasible)

**Fig. 1.**
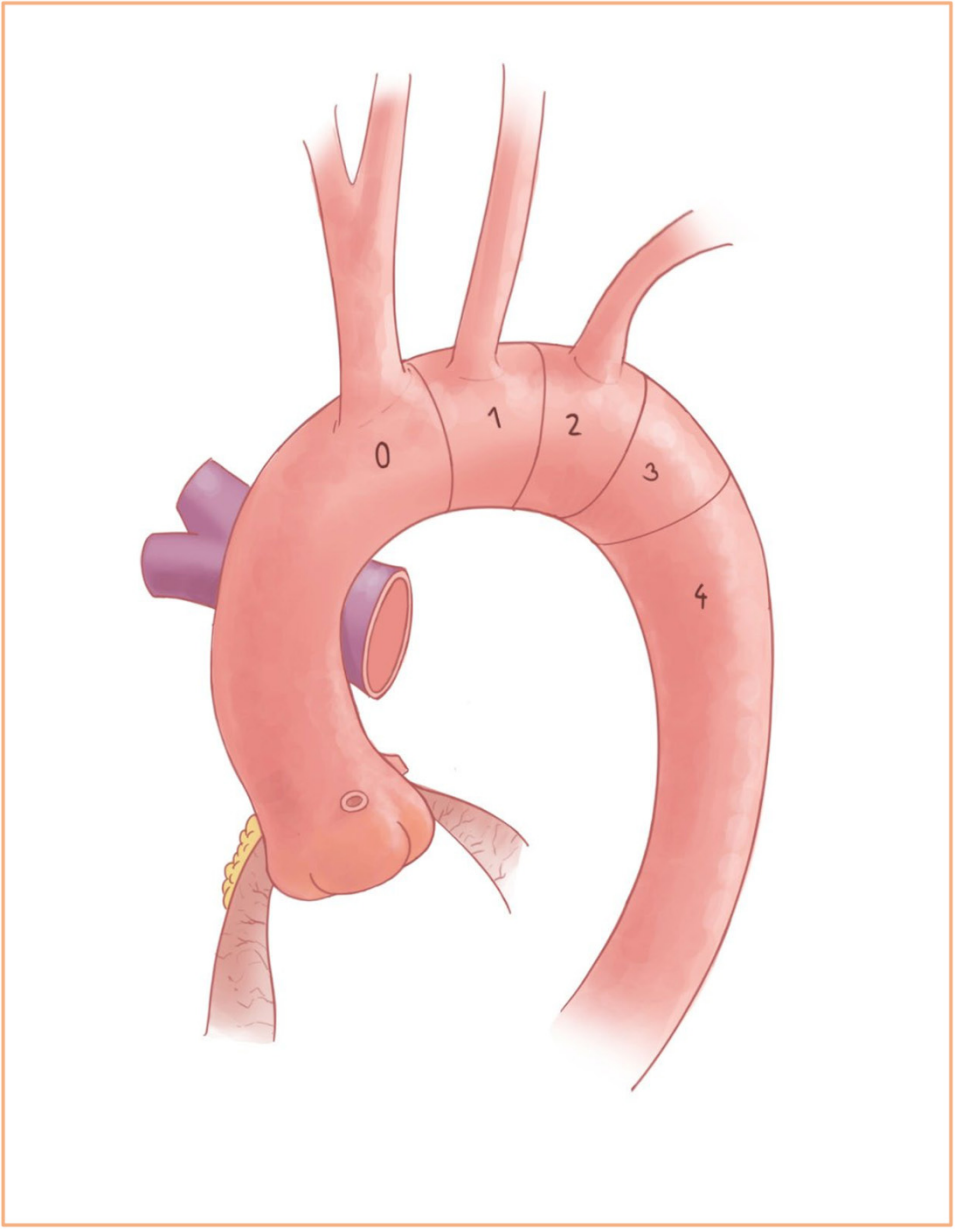
The aortic arch with the corresponding attachment zones.

**Fig. 2.**
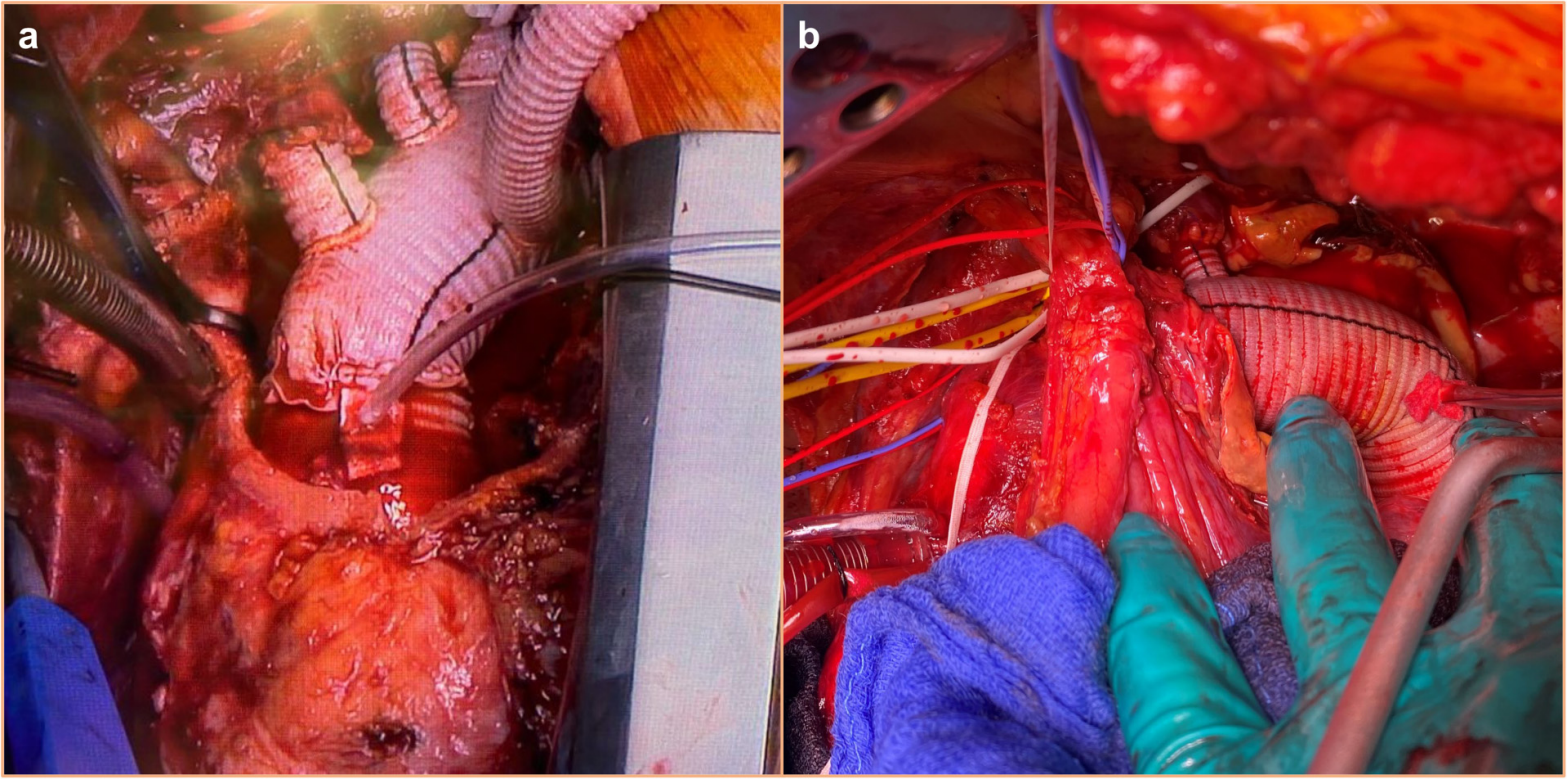
Examples of open aortic arch repair concepts. (a) Total arch replacement with selective reimplantation of the supra-aortic vessels. (b) Distal aortic arch replacement with selective reimplantation of the left subclavian artery.

The extent of open arch repair is determined by the extent of the indicative pathology (Fig. 1). Patients with aneurysms including the complete aortic arch (with acceptable surgical risk) and Stanford type A dissections with (re)entries on the outer arch curvature or at the origin of the supra-aortic vessels generally require an open total arch replacement (TAR; Fig. 2a). TAR can be performed with selective reimplantation of the supra-aortic vessels or, in the case of heavy calcifications at the origin of the branches, with an island reimplantation technique. When operating on patients with known genetic disorders such as Marfan syndrome or Loeys–Dietz, one should avoid the island reimplantation technique to avoid residual aortic tissue. When performing TAR, downstream aortic pathology techniques, mainly the elephant trunk or the frozen elephant trunk (FET) technique, might be required.

**Fig. 3.**
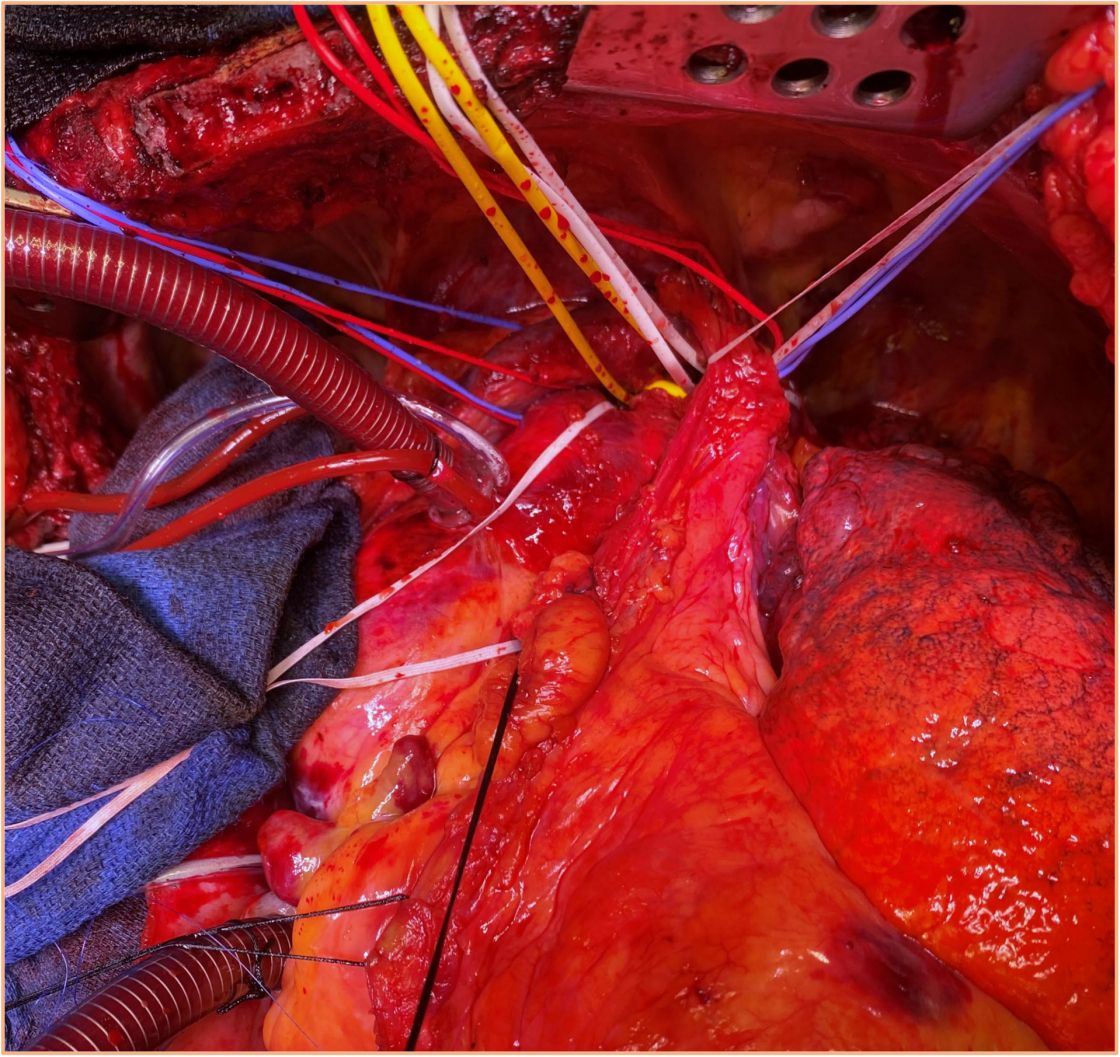
Surgical exposure of the complete aortic arch via a bilateral clamshell incision for treatment of distal aortic arch procedures.

Not always does the maximum option, TAR, constitute the preferable option for the patient. When treating ascending aortic aneurysms that continue into zone 1 or 2, either an aggressive hemiarch or a partial arch replacement (i.e., zone 1 or zone 2 arch repair) should be considered. Partial arch repair brings the distal anastomosis further upstream and therefore shortens the circulatory arrest time and provides better hemostatic control. An important factor for successful performance of partial arch repair is the tissue quality at the level of the distal anastomosis. In case of a thin aortic wall, excessive calcifications, or large areas of soft plaques in zone 1 or zone 2, a TAR might be the better choice (given a superior aortic tissue quality in zone 3). When operating on patients with Stanford type A dissections with involvement of the arch with only a single entry at the inner curvature, an aggressive hemiarch with exclusion of the entry might suffice and obviates the need for a patient to undergo a longer and more complex procedure in an acute setting. In rare cases in which only a distal arch pathology is present (aneurysm, penetrating aortic ulcer, calcified coarctation in the adult), a partial replacement of the distal arch (and the descending aorta) should be considered (Fig. 2b). In those cases, median sternotomy might be abandoned as main access in favor of a clamshell or a left lateral thoracotomy (Fig. 3). In rare cases of circumscribed disease processes such as aortic ulcers in good accessible position (anterior on the smaller curvature), a pericardial patch plasty with a short circulatory arrest and no prosthetic replacement of the arch might be the preferred type of open arch repair.

## 4. Respect the Distal Aortic Anastomosis

Distal aortic anastomosis is arguably the most challenging in aortic arch replacement. It is localized deep posterior in the thoracic cavity, the angles are demanding, the exposure is difficult, and delicate structures (recurrent laryngeal nerve, phrenic nerve) are in close proximity. Hemostatic suturing is of utmost importance, as there is only one chance for corrective sutures, which is before reimplantation of the supra-aortic vessels. The assistant needs to provide good exposure of the aortic wall to allow exact placement of every stitch. In cases of aortic dissection, a sandwich should be performed with 2 felt strips (outside and inside the native aortic wall) and a 4–0 polypropylene suture using a running mattress technique. We frequently use bovine pericardial strips instead, as we believe that they possess superior hemostatic qualities. However, suturing with these strips is likely more cumbersome because of their floppy texture. Subsequently, the prosthetic anastomosis can be performed using a 4–0 polypropylene suture in a continuous running technique. When performing the anastomosis in nondissection patients, in whom no sandwich is required, an autologous pericardial strip can be incorporated in the suture line for hemostatic reasons. Subsequently, we reinforce the anastomosis with approximately 6 to 8 interrupted patch u-sutures in a circular fashion. Following completion, exact deairing is done, and antegrade flow should be reestablished over the designated prosthetic side branch. Now is the time for a meticulous assessment of the anastomosis. If there is bleeding, it should be addressed immediately, as exposure will be very limited later, and severe bleeding from this location can have fatal consequences.

## 5. Do Not Shy Away from Hybrid Procedures

When offering open aortic arch repair, the possibility of hybrid procedures must be kept in mind. Although hybrid procedures should not be considered less invasive, as they often include cardiopulmonary bypass and circulatory arrest, they ideally can (1) facilitate open repair and (2) allow a more comprehensive addressing of the pathology. A promising hybrid concept for functional TAR that fulfills both mentioned criteria is the zone 2 aortic arch repair.^14^ The distal anastomosis is sutured in zone 2, and the carotid and innominate arteries are selectively reimplanted. This allows the proximalization of the distal anastomosis, which is advantageous for stated reasons, ergo facilitating open repair. In addition, the proximalization allows for an adequate landing zone between the left carotid branch and the in situ left subclavian artery for further stent graft deployment, thus allowing a more comprehensive solution. This concept might prove beneficial in cases with unfavorable anatomy, low-quality aortic tissue, or severe aortic calcifications in zone 3. Different strategies that include debranching of the supra-aortic vessels offer similar advantages, including proximalization of the distal anastomosis and improved landing zones for further endovascular aortic repair of the distal arch and the descending aorta. A true minimally invasive hybrid approach, which omits cardiopulmonary bypass and circulatory arrest, is the combination of carotid subclavian bypass for creation of a landing zone and subsequent thoracic endovascular aortic repair (TEVAR). This approach provides a good alternative for patients with distal arch or downstream aortic pathology and is ideally performed in a hybrid setting by members of the aortic team.

## 6. Know Your Armamentarium

**Fig. 4.**
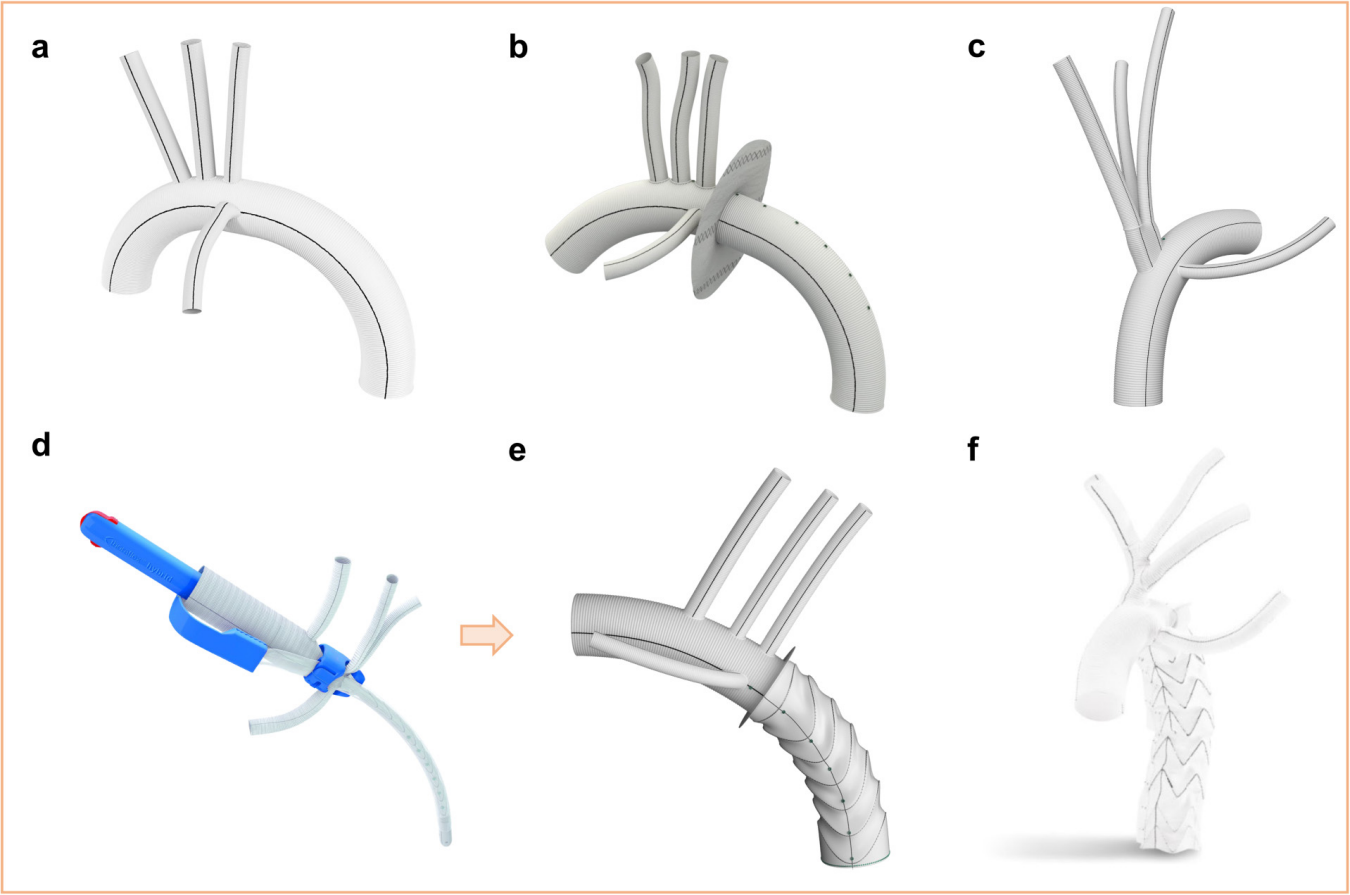
Overview of currently available aortic arch protheses available for TAR. (a) Gelweave Plexus prosthesis (Terumo Aortic, Inchinnan, UK) with 3 side branches for vessel reimplantation. (b) Gelweave Siena prosthesis (Terumo Aortic) with an integrated collar for size mismatch at the distal aortic anastomosis. (c) Gelweave Lupiae prosthesis (Terumo Aortic) with a proximal trifurcated graft for reimplantation after debranching of the supra-aortic vessels. (d) Delivery system of the Thoraflex Hybrid prosthesis (Terumo Aortic) for TAR with the FET technique. (e) Thoraflex Hybrid prosthesis with expanded stent graft. (f) E-vita OPEN NEO (Artivion, Inc., Kennesaw, GA, USA) for TAR with FET, which features a trifurcated graft for vessel reimplantation after debranching (*used with the permission of Artivion, Inc.*). FET, frozen elephant trunk; TAR, total arch replacement.

A variety of aortic arch prosthetics are available with distinct design features (Fig. 4). When performing prosthetic arch replacement, the surgeon needs to know the available options and how to make use of them. For classic TAR with selective reimplantation of the supra-aortic vessels without further downstream adjuncts (ET or FET), the Gelweave^TM^ Plexus (Terumo Aortic, Inchinnan, UK) is generally considered our primary choice; it consists of an arched main graft with 3 side branches for reimplantation and 1 perfusion branch and is available in 2 mm increments from 20 to 34 mm sizes (Fig. 4a). In case of an expected size mismatch at the distal anastomosis due to residual aortic aneurysm, the Gelweave Siena prosthesis (Terumo Aortic), an iteration of the Plexus prosthesis, offers practicable design features (Fig. 4b). This provides similar vessel and perfusion branches as the Plexus prosthesis and features a specific collar at the designated location of the distal anastomosis to compensate for an expected aortic graft mismatch. In addition, radiopaque markers are provided in the distal graft as fluoroscopic landmarks for subsequent endovascular repair. Another prosthesis that can be applied in hybrid procedures is the Gelweave Lupiae prosthesis (Terumo Aortic), which features a proximal trifurcated graft for vessel reimplantation with a radiopaque marker at its base and a perfusion side branch (Fig. 4c). For patients requiring TAR with the FET technique, the Thoraflex^TM^ Hybrid (Terumo Aortic) offers a reliable platform (Fig. 4d–e); it consists of a multibranched arch prosthesis (3 branches for vessel reimplantation, 1 perfusion side branch) that incorporates an aortic Relay Pro stent, which is deployed in open antegrade fashion. A collar is integrated distally to the perfusion branch at the location of the distal aortic anastomosis. The E-vita OPEN NEO (Artivion, Inc., Kennesaw, GA, USA) is another hybrid prosthesis that supports the FET technique. It exists in a classical 3 side-branch version and, as currently the only FET prosthesis, with a trifurcated proximal graft (Fig. 4f). All models come with a collar at the designated distal aortic anastomosis location and a perfusion side branch. The E-vita OPEN NEO is not impregnated with gelatin or collagen; therefore, increased prosthetic oozing might occur. Contradicting reports of bleeding complications following surgery with hypothermic circulatory arrest are available in the literature.^15,16^

## 7. Choose an Adequate Perfusion Concept

The right cannulation strategy is a key part of open aortic arch repair, and several concepts may be feasible in certain situations. We moved away from femoral and direct aortic cannulation to right axillary artery cannulation as the standard approach in classical TAR, because it facilitates the application of antegrade cerebral perfusion. To avoid limb ischemia, an 8 mm Dacron prosthesis is anastomosed in end-to-side fashion to the axillary artery. The arterial portion distal to the graft is snared with a vessel loop, and tension can be applied to regulate the mean arterial pressure and avoid hyperperfusion of the arm. Although no randomized trials exist, evidence supports the use of axillary artery cannulation for proximal thoracic aortic procedures.^17,18^ In case of a long innominate artery with good tissue quality, cannulation of the innominate can be performed in the same fashion as the axillary artery. This avoids a second incision and avoids complications such as brachial nerve plexus injuries. Femoral artery cannulation, open or percutaneous, is generally reserved for hemodynamically unstable patients who require urgent mechanical circulatory support or as a bail-out strategy. When operating on a distal aortic arch pathology (and clamping between zone 1 and zone 2 is possible) a standard cannulation of the ascending aorta (for supra-aortic vessel perfusion) in combination with femoral cannulation (for retrograde visceral perfusion) presents a viable option (Fig. 3).

## 8. Provide Adequate Cerebral Protection

Perioperative stroke is one of the most dreadful and feared adverse events of open arch repair, and great care should be taken to prevent it. The first report of deep hypothermic circulatory arrest for arch replacement by Griepp and colleagues represented a milestone in neuroprotection for arch surgery.^5^ Since then, utilization of various degrees of systemic hypothermia has become standard in almost every arch procedure, depending on the use of concomitant retrograde or selective ACP. We advocate the combination of moderate hypothermia (26 °C to 28 °C) in combination with bilateral selective ACP. Evidence supports the use of this regimen over stand-alone deep hypothermic circulatory arrest with reduced rates of perioperative stroke.^19,20^ Although retrograde cerebral perfusion is used by some centers, it does not provide adequate cerebral perfusion on the capillary level, and its benefits, besides maintaining cerebral hypothermia, remain questionable.^21^ When performing open arch repair with cannulation of the right axillary or innominate artery, clamping of the innominate provides unilateral ACP. Depending on the anatomy of the circle of Willis and the intracerebral collateralization, this might already provide sufficient oxygenation of both hemispheres. Nevertheless, we always aim to establish bilateral ACP, as it provides a possible survival benefit in circulatory arrest times of greater than 50 min.^22^ If feasible, perfusion of the left subclavian artery prevents overt backflow and adds protection of the left vertebral artery.

## 9. Respect the Hemostatic Management

When performing prosthetic open arch replacement under hypothermic circulatory arrest, bleeding remains an issue and must be addressed properly in all stages of the procedure. If one has the possibility to opt for a moderate hypothermic approach with ACP, this might decrease bleeding compared with deep hypothermic arrest.^23^ Furthermore, meticulous assessment of every anastomosis immediately after completion is of utmost importance, as there might not be an opportunity to access them in later stages of the procedure. After weaning of cardiopulmonary bypass and administration of protamine, one should actively look for bleeding at all anastomoses. Some manipulation of the graft and vessels might be needed to identify hidden bleeding sites. In parallel, the anesthesiologist must actively support hemostasis with an adequate pharmacologic regimen. To substitute properly and not blindly, monitoring or the activated clotting time for prolonged heparin effects is mandatory. Bedside coagulation and platelet function tests such as thromboelastography (TEG^®^, Haemonetics, Boston, MA, USA) or rotational thromboelastometry (ROTEM^®^, Werfen, Barcelona, Spain) are very useful instruments that provide valuable onsite information about clot formation and dissolution after cardiopulmonary bypass. After 15 to 30 min, they allow for specific substitution of platelets, coagulation factors, or fibrinogen in the surgical theater. Coagulation needs to be actively monitored, not only in the surgical theater but also in the intensive care ward to substitute platelets, coagulation factors, or protamine early and prevent postoperative bleeding.

## 10. Consider Endovascular Concepts

The success of open arch repair is determined by the appropriate patient selection and should be done by the aortic team in aortic board meetings (see the first commandment). When the patient presents with prohibitive risk for open arch repair, which remains the gold standard,^24^ TEVAR represents a promising treatment alternative. Although no absolute criteria for either open or endovascular repair exist, several factors need to be considered to tailor patient-specific concepts. The presence of connective tissue disorders, small vascular access (<7 mm), native ascending diameters greater than 38 mm, and concomitant heart valve disease favor an open repair, whereas severely impaired left or right ventricular function, previous coronary artery bypass grafting with a patent left internal mammary at risk with resternotomy, and severely impaired pulmonary or liver function favor an endovascular concept.^12^ When deciding on endovascular repair, 2 main options for stent graft devices are currently available: branched stent grafts (bTEVAR) and fenestrated stent grafts (fTEVAR). Both present with favorable technical success rates and different limitations regarding patient anatomy and indication. Currently, bTEVAR seems to offer a broader applicability, although it is associated with higher rates of stroke compared with fEVAR.^25^

## Conclusions

Due to advancements in neuroprotection, open aortic arch repair has become a standardized procedure, and favorable outcomes can be achieved when performed in expert centers. Modern hybrid concepts expanded the application to a wider patient spectrum. Nonetheless, it remains a highly complex procedure with potentially severe complications. Therefore, careful patient selection, meticulous surgical preparation, and rigorous execution are imperative.
